# The United States Food and Drug Administration and Prescription Drug Promotion

**DOI:** 10.19102/icrm.2019.100305

**Published:** 2019-03-15

**Authors:** Ankur S. Kalola, Robert Dean

**Affiliations:** ^1^Office of Prescription Drug Promotion, United States Food and Drug Administration, Silver Spring, MD, USA

**Keywords:** Advertising, Food and Drug Administration, Office of Prescription Drug Promotion, prescription drug promotion, promotion

## Abstract

The impact of prescription drug promotion on health-care professionals (HCPs) is significant. Pharmaceutical industry spending on promotion to HCPs greatly outpaces spending on direct-to-consumer promotion. According to Syneos Health™ PromotionalAnswers, in 2017, the pharmaceutical industry spent more than $24 billion on drug promotion, with more than $18.5 billion allotted for marketing to HCPs. Although prescription drug promotion can provide valuable information about drug therapies, it is essential that it be truthful, balanced, and not misleading, because HCPs may consider this information when making treatment decisions for their patients.

## The United States Food and Drug Administration and prescription drug promotion

The impact of prescription drug promotion on health-care professionals (HCPs) is significant. Pharmaceutical industry spending on promotion to HCPs greatly outpaces spending on direct-to-consumer promotion. According to Syneos Health™ PromotionalAnswers, in 2017, the pharmaceutical industry spent more than $24 billion on drug promotion, with more than $18.5 billion allotted for marketing to HCPs.^1^ Although prescription drug promotion can provide valuable information about drug therapies, it is essential that it be truthful, balanced, and not misleading, because HCPs may consider this information when making treatment decisions for their patients.

### What laws govern prescription drug promotion?

Under the Federal Food, Drug, and Cosmetic Act (FD&C Act) of 1938, the United States Food and Drug Administration (FDA) is responsible for regulating the manufacture, sale, and distribution of drugs in the United States. This authority includes oversight of the labeling of drugs and the advertising of prescription drugs.^a^ The law and its implementing regulations require that advertisements (ads) and labeling for prescription drugs be truthful, balanced, and not misleading. If promotional materials for a drug are found to be false or misleading in any particular manner, then that drug is misbranded according to the FD&C Act and may be subject to enforcement action.

## Who is responsible for regulating prescription drug promotion?

As part of the FDA, the Office of Prescription Drug Promotion (OPDP) is charged with protecting the public health by regulating prescription drug promotion. The OPDP resides within the Center for Drug Evaluation and Research, which reviews and approves prescription drugs.^b^

### What are the roles of the Office of Prescription Drug Promotion?

The OPDP is responsible for ensuring that the information contained in prescription drug advertising and promotional labeling is truthful, balanced, and not misleading. The OPDP engages in a variety of tasks to perform this responsibility, including

- Providing written advisory comments to drug companies on proposed promotional materials
- Reviewing complaints about alleged violations
- Sending Untitled or Warning letters to notify firms of allegedly false or misleading promotional materials for the purpose of obtaining voluntary compliance
- Comparing the product labeling and promotional materials of various closely related products to ensure that the regulatory requirements are consistently and equitably applied
- Attending major medical meetings and pharmaceutical conventions to monitor promotional exhibits and activities
- Acting as a liaison with other FDA divisions on the subject of promotional issues

### What types of promotion does the Office of Prescription Drug Promotion regulate?

The OPDP is responsible for promotional labeling and advertising, which are both used to help sell prescription drugs.

Prescription drug advertising includes ads placed in magazines, journals, periodicals, and newspapers as well as those broadcast on television, radio, and by way of telephone communication systems.

Prescription drug promotional labeling includes labeling other than FDA-required labeling that generally is devised for the promotion of a drug product and is distributed by the manufacturer, distributor, packer, or any party acting on the sponsor’s behalf. Examples of this can include brochures, sales aids, mailing pieces, and slide decks.

### What types of promotion does the Office of Prescription Drug Promotion not regulate?

The OPDP does not regulate the promotion of over-the-counter drugs, drugs for animals, dietary supplements, devices, certain biological products, foods, or cosmetics. Promotion of these products is regulated by other centers in the FDA or by the Federal Trade Commission (FTC).

In addition, the OPDP does not regulate truly independent and nonpromotional industry-supported scientific and educational activities. This could include a continuing medical education program that is supported by a drug company but that is nonpromotional in nature and otherwise independent from the substantive influence of the supporting company.^2^

## What are some different types of promotional materials?

### Product claim

The most common type of promotional materials utilized by sponsors, product claim materials, include the name of a drug, information about the approved indication, and the drug’s benefits and risks. Examples include sales aids, journal ads, television ads, slide decks, product websites, and mailing pieces.

Product claim materials include the name of the drug [ie, proprietary (brand) and established (generic)], at least one FDA-approved use for the drug, and the most significant risks of the drug. The risks and benefits are also required to be presented in a balanced fashion. Balance depends on both the content of the information in the promotional materials and how the information is presented.

### Reminder

These types of promotional materials include the proprietary and established names of a drug. They also can include the dosage form; package contents; price; and the name of manufacturer, packer, or distributor. They do not include indications, dosage recommendations for use, or other written, printed, or graphic representations or suggestions related to the drug product. Reminder ads are not required to include risk information. Notably, reminder promotion is not permitted for drugs with boxed warnings. These types of warnings are also commonly referred to as “black box warnings.” They appear on a prescription drug’s label and are designed to call attention to serious or life-threatening risks.

### Institutional

These types of promotional materials include information such as the drug company name and area of research but do not mention any specific drug names. These promotional materials are not prescription drug promotion materials subject to FDA regulation, but may be subject to regulation by other government agencies such as the FTC.

### Help-seeking/disease awareness

These types of promotional materials discuss a medical condition or disease state and can include a drug company name and telephone number to call for more information; however, they do not recommend or suggest a specific drug treatment. These promotional materials are also not prescription drug promotion subject to FDA regulation, but may be subject to such by other government agencies including the FTC.

### Coming soon

These types of promotional materials announce the name of a soon-to-be-approved new drug product. When disseminated by a sponsor or investigator (or someone acting on their behalf), such promotional materials must not represent that the drug is safe or effective for the purposes for which it is still being investigated.

## Does the Office of Prescription Drug Promotion “approve” or “preclear” prescription drug promotional material before use?

The OPDP does not approve or preclear promotional materials before they are used. For most products, drug companies are only required to submit their promotional materials to the OPDP when they first appear in public, meaning that the OPDP sees many promotional materials at about the same time as the public does. Thus, the public may see promotional materials that violate the law, resulting in misbranded products, before the OPDP can stop them from appearing or seek corrections to the materials.

However, the OPDP does provide opinions on proposed promotional materials before they are used upon voluntary request by a drug company. For example, drug companies often voluntarily seek advice from the OPDP when a new product is approved or before they release a television broadcast ad.

## What could make promotional materials false or misleading?

There are a number of ways in which promotional materials may violate the advertising and labeling regulations. For example, the promotional material could

- Make claims that are not adequately supported
- Misrepresent data from studies
- Overstate the drug’s benefits
- Omit or downplay important risk information
- Promote an investigational drug as safe and effective for the use(s) under investigation, when the drug has not been proven to be safe and effective within the meaning of the FD&C Act and has not been approved as a drug under that authority for any use
- Omit material facts about the drug
- Fail to present a “fair balance” of information relating to the drug’s risks and benefits

## How can you report potentially false or misleading promotion?

In 2010, the OPDP launched the Bad Ad outreach program to encourage HCPs to recognize and report potentially false or misleading prescription drug promotional material. The program helps HCPs (including physicians, nurse practitioners, nurses, physician assistants, and pharmacists) who prescribe medications or otherwise make selection decisions about the use of prescription drugs for patients to better understand what constitutes appropriate prescription drug promotion and how to report possible violations.

The OPDP has created a dedicated toll-free number and email address for HCPs to communicate their concerns to the OPDP or report potentially false or misleading prescription drug promotion. Contact information for the Bad Ad Program is as follows: toll-free 855-RX-BADAD (phone) or BadAd@fda.gov (email).

## Frequently asked questions regarding the Bad Ad program

### Can I make a report anonymously?

Yes, anonymous complaints often alert the OPDP to potential problems. However, complaints accompanied by names and contact information are helpful in cases in which the OPDP must conduct further follow-up for more information.

### Will the OPDP be able to stop the misleading promotion?

In many cases, yes, especially if evidence is provided (eg, a copy of the promotional material).

### What will happen to my complaint once I have contacted the OPDP?

The information you provide will be sent to an OPDP reviewer. The reviewer will evaluate it and determine if it provides information that is valuable for our ongoing surveillance activities or if it even supports sending the company an Untitled or Warning letter. These letters notify a company of an alleged violation and request that the company cease the activity that gave rise to the alleged violation and, in the case of the latter, request that the company take steps to correct the issue(s) cited in the letter. If a firm fails to come into compliance with the law, the FDA may initiate a seizure or injunction action or a criminal investigation.

### How do I learn more?

To request an OPDP in-service training event for a large medical group/hospital or to speak directly with an OPDP reviewer, call 301-796-1200.

## Footnotes

^a^See 21 U.S.C. 352(a); 21 U.S.C. 352(n). The term “labeling” has been interpreted broadly by the U.S. Supreme Court to include materials that supplement or explain an article. *Kordel v. United States*, 355 U.S. 345 (1948) (holding that the term “accompanying such article” does not restrict the definition of labeling to materials that physically accompany the article). No physical attachment is necessary between the materials and the article; rather, the textual relationship of the items will determine whether the printed, written, or graphic material constitutes labeling. This includes both FDA-required labeling and promotional labeling.

^b^For certain biological products that are regulated as drugs, such as vaccines, the Advertising and Promotional Labeling Branch of the Center for Biologics Evaluation and Research is responsible for regulating promotion.
